# Case Report: Immune checkpoint inhibitor-induced myositis/myocarditis with myasthenia gravis-like symptoms in a colon cancer patient with suspected thymic metastasis

**DOI:** 10.3389/fonc.2026.1808170

**Published:** 2026-08-11

**Authors:** Xiao Zhang, Hui-Li Wang, Kai-Lun Liu, Xin-Hua Zhang, Gui-Juan Wang, Xue-Qi He, Qin Du, Hui Li, Li-Na Feng, Chang-Lun Li, Dong Yang, Wen-Juan Zheng, Lei Han, Peng Li

**Affiliations:** 1 Department of Hepatobiliary Surgery, Affiliated Hospital of Jining Medical University, Jining, Shandong, China; 2 Department of Medical Oncology, Affiliated Hospital of Jining Medical University, Jining, Shandong, China

**Keywords:** colon cancer, immune checkpoint inhibitors, myasthenia gravis, myocarditis, myositis

## Abstract

**Background:**

The Widespread application of immune checkpoint inhibitors (ICIs) has led to a noticeable increase in patients developing the triad of severe myasthenia gravis (MG), myocarditis, and myositis. However, this overlapping triad syndrome is still very rare, characterized by a subtle onset, a rapid progression, and a high mortality rate. Due to its insidious onset and diagnostic challenges, there is currently limited knowledge of this overlapping triad syndrome in clinical settings.

**Case presentation:**

We report the case of a male colon cancer patient with suspected thymic metastasis who developed the overlapping immune-related myocarditis, myositis, and MG-like symptoms after one cycle of ICI treatment. The patient received timely administration of corticosteroids, oxygen therapy, immunoglobulin, and other treatments, following which he recovered and was discharged. No discernible adverse impact on his subsequent quality of life was observed.

**Conclusions:**

Vigilance is warranted for the risk of developing a triad of severe MG, myocarditis, and myositis when patients receiving ICIs present with symptoms such as exertional dyspnea, swallowing weakness, diplopia, dysarthria, limb weakness, or inability to walk due to muscle pain, particularly for patients with thymic masses. Such patients should be monitored in the hospital and, if necessary, admitted to the intensive care unit, where they will be managed by a multidisciplinary team of specialists.

## Introduction

Immune Checkpoint Inhibitors (ICIs) is a class of monoclonal antibodies that target negative regulatory factors in T-cell activation. By targeting overactive inhibitory T-cell pathways, they enhance the activity of anti-tumor T cells, thereby killing tumor cells (1). Since the US Food and Drug Administration approved the first ICI, ipilimumab, for the treatment of melanoma in May 2011, significant progress has been made in the research and development of ICIs. Currently, ICIs have been widely used in the treatment of various malignant tumors, significantly improving the prognosis of many types of malignancies, including metastatic melanoma, non-small cell lung cancer, renal cell carcinoma, and others, revamping the landscape of tumor treatment (2–4). However, the immune - related adverse events (irAEs) caused by ICIs have become a new challenge in clinical management.

Among the irAEs caused by ICIs, the MMM overlapping syndrome of myositis, myocarditis, and myasthenia gravis (MG) is particularly dangerous, with a mortality rate as high as 30% - 50% (5). This is caused by ICIs, which activate T-cell-mediated autoimmune responses that simultaneously target the neuromuscular junction, myocardium, and skeletal muscles. It is reported that this overlapping syndrome is more likely to occur in patients with thymic tumors (6), and relevant cases have also been reported in renal cancer, bladder cancer, gastric cancer, and liver cancer (7–10). However, relevant cases in colon cancer patients are still extremely rare. Additionally, the early manifestations of this type of overlapping syndrome are often atypical and easily confused with tumor progression or infection, leading to treatment delays (11). Therefore, a full understanding of this immune-related adverse reaction is of great clinical significance for the clinical application of ICIs.

This paper reports a colon cancer patient with suspected thymic metastasis who developed myositis, myocarditis, and MG-like symptoms after ICI treatment and discusses the treatment process. This helps deepen clinicians’ understanding of ICI- related irAEs and provides a new clinical perspective for optimizing the risk management of immunotherapy in cancer patients.

## Case report

**Figure 1:**
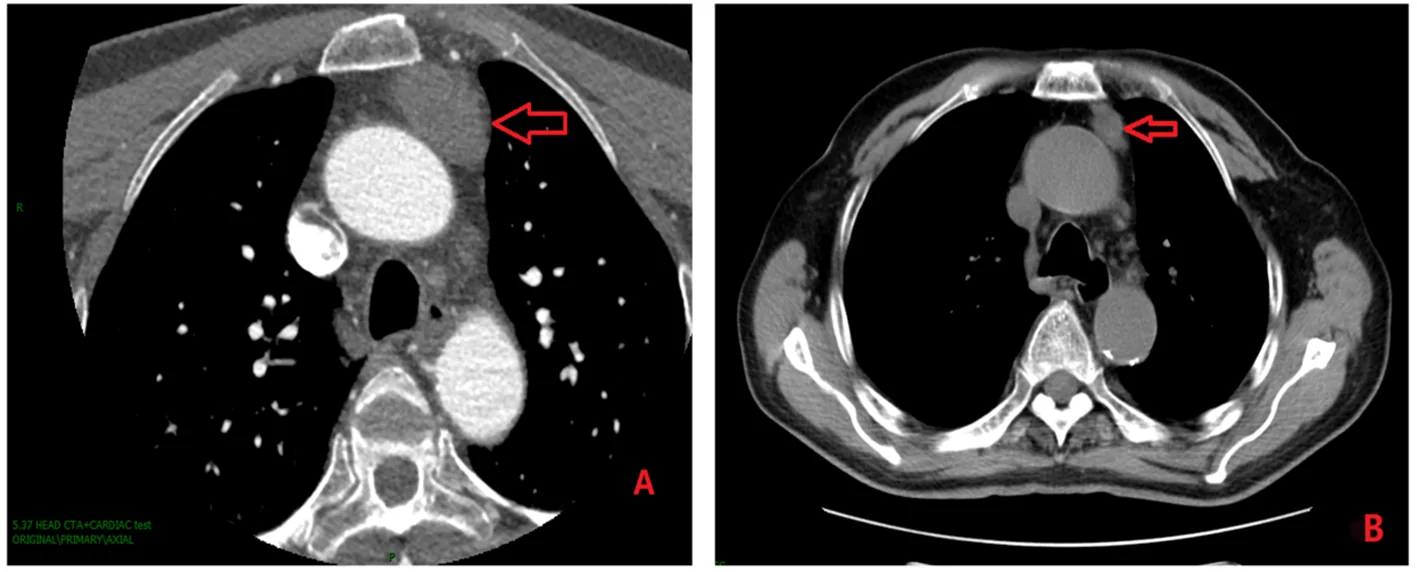
Chest computed tomography (CT) images of the patient before and after treatment with sintilimab. **(A)** CT image on March 5, 2022 before treatment with sintilimab. **(B)** CT image on May 28, 2022 after treatment with sintilimab. Lesions in the upper mediastinal soft tissue were indicated by arrows.

**Figure 2:**
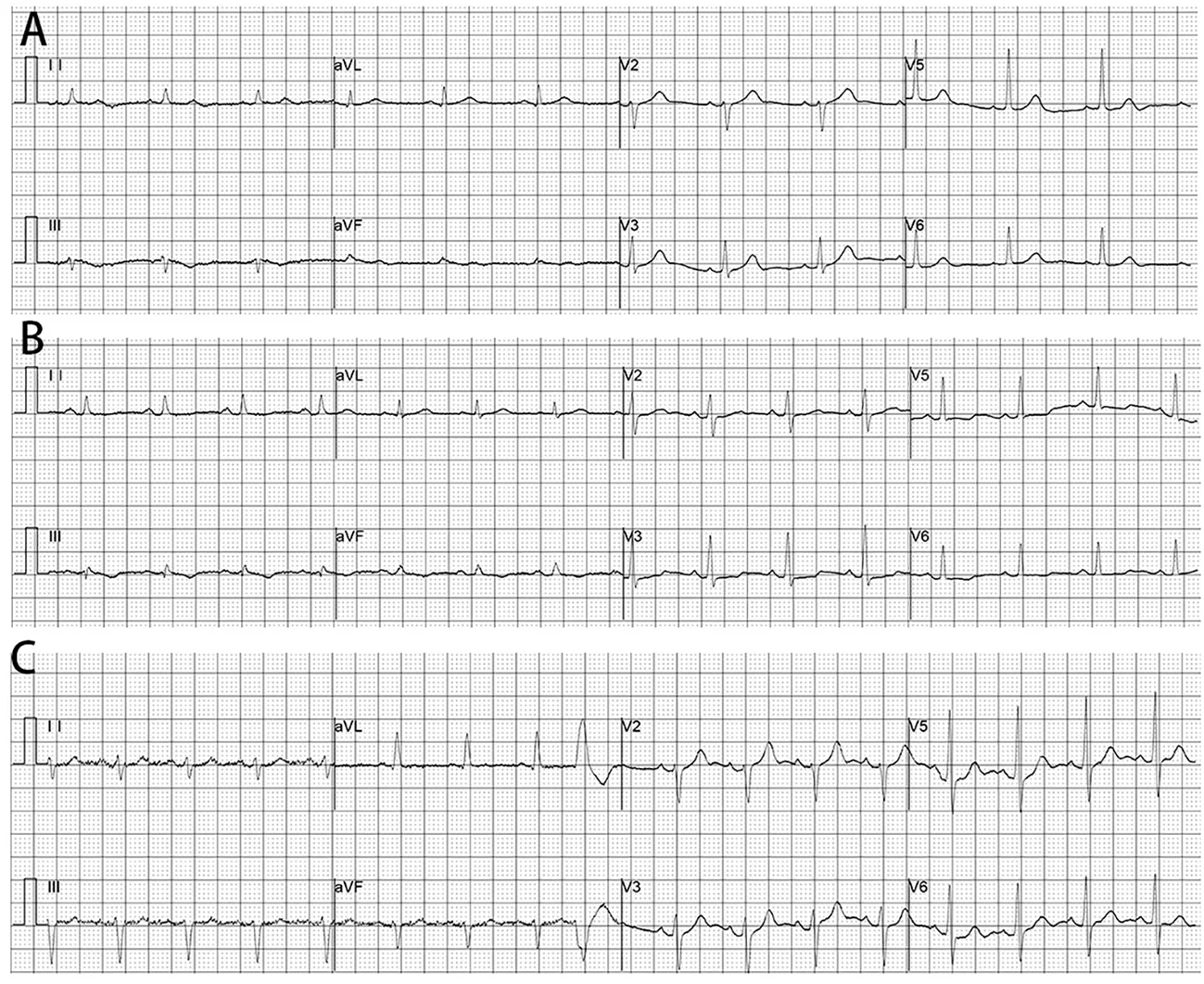
The dynamic changes of electrocardiogram (ECG). The patient’s initial ECG was approximately normal upon admission, followed by anomalies such as low voltage in limb leads causing a QRS complex, sinus tachycardia, ventricular premature complex(es), ST deviation, and T-wave abnormality. (Papre speed: 25mm/s Sensitivity:10mm/mv). **(A)** April 19, 2022 ECG Interpretation: Approximately normal ECG. **(B)** April 23, 2022 ECG Interpretation:1. Sinus rhythm 2. Low voltage in limb leads causing QRS complex 3. V2 R/S ratio > 1 4. T-wave changes. **(C)** May 9, 2022 ECG Interpretation: 1. Sinus tachycardia 2. Left axis deviation 3. Premature ventricular contractions 4. rs pattern in leads III and aVF 5. Mild ST-segment changes (please consider clinical analysis).

In February 2022, a 72-year-old man presented with upper abdominal pain and was admitted to the Affiliated Hospital of Jining Medical University. Colonoscopy examination showed the presence of colon adenocarcinoma, which was located approximately 60 cm from the anus, along with (transverse) colon cancer. Subsequently, a thoracoabdominal computed tomography (CT) scan detected a thymic mass, and the possibility of metastasis could not be excluded. Additionally, small pulmonary nodules were found, with the potential to be metastatic lesions (**Figure 1A**). PET-CT was not performed due to its high cost and the patient’s limited family income. Due to severe intestinal obstruction, the patient underwent palliative resection of the transverse colon without colostomy. Intraoperative findings confirmed transverse colon cancer with incomplete obstruction. Given that preoperative evaluation could not rule out pulmonary or mediastinal metastasis, palliative resection was performed. The procedure was as follows: the incision was protected, and the small intestine was packed into the left upper abdomen with gauze rolls. The gastrocolic ligament was divided, and the transverse colon was mobilized. The hepatic flexure was dissected upward to the predetermined transection line approximately 10 cm from the tumor. The middle colic vessels were clamped and ligated, and lymph nodes at the vascular root were dissected. The splenic flexure was mobilized to the predetermined transection line approximately 10 cm from the tumor. After colonic transection, the stump was closed with a purse-string suture, and the anvil of a 31-mm circular stapler was inserted and secured. The transverse colon was incised approximately 10 cm distal to the tumor, and the stapler body was introduced. The central rod was passed through an avascular area of the lateral wall of the transverse colon, connected to the anvil, and fired after tightening, achieving an end-to-side transverse colonic anastomosis. The anastomosis was explored and confirmed to be patent and hemostatically satisfactory. Finally, the mesocolic defect was closed with sutures. Post-operative pathological examination indicated moderately to poorly differentiated adenocarcinoma in the transverse colon, with the tumor measuring 8.5 × 6 × 3 cm. The cancer had invaded the entire layer of the intestinal wall, extended into the subserosal adipose tissue, and had evidence of nerve invasion. However, no definite intravascular tumor emboli were observed. Moreover, no metastatic cancer was detected in the pericolonic lymph nodes (0 out of 12 nodes were positive). Next-Generation Sequencing (NGS) revealed deficient mismatch repair/high microsatellite instability (MSI-H) status. After the palliative surgery, the patient’s post-operative condition was satisfactory, and he was diagnosed with advanced-stage colon cancer with suspected lung and mediastinal metastases. On March 29, 2022, the patient received a treatment cycle in the Department of Oncology. On the first day, he was administered a single injection of sintilimab [Innovent Biologics (Suzhou) Co., Ltd., drug registration number: S20180016, specification: 100 mg (10 mL)/vial] at a dose of 200 mg. In addition, capecitabine [Qilu Pharmaceutical Co., Ltd., drug registration number: H20133361] was prescribed at a dose of 2.0 g twice daily from days 1 to 14, with a treatment cycle of every 3 weeks. On April 19, 2022, when the patient returned for the second cycle of treatment, blood tests showed elevated cardiac enzymes and transaminase. The creatine kinase MB isoenzyme level was 108.08 ng/mL, the alanine aminotransferase (ALT) activity was 90.1 U/L, and the aspartate aminotransferase (AST) activity was 263 U/L. Cardiac injury marker tests indicated a creatine kinase isoenzyme level of 178.8 ng/mL, a myoglobin level of 1996.70 ng/mL, and a high-sensitivity troponin I level of 0.3397 ng/mL. The detailed laboratory parameters and tumor marker results are presented in Supplementary Material 1. However, electrocardiography (ECG) did not show any significant abnormalities (**Figure 2A**).

After consulting with the cardiology department, echocardiography and cardiac magnetic resonance imaging (MRI) were recommended. Additionally, oral coenzyme Q10 was prescribed for cardiac nutrition. However, the patient’s family refused further hospitalization and requested discharge. Subsequently, on April 22, 2022, the patient began to experience muscle pain and discomfort in both lower limbs and the back. On April 23, 2022, cardiac enzyme panel tests conducted during an outpatient visit indicated a sustained increase in creatine kinase, creatine kinase isoenzyme MB, and aminotransferase levels compared to previous measurements. Specifically, the creatine kinase level reached 14,465 U/L, and the creatine kinase isoenzyme MB level was 202.01 ng/ml. The ECG also showed notable changes:1. Sinus rhythm 2. Low voltage in limb leads causing QRS complex 3. V2 R/S ratio > 1 4. T-wave changes (please consider the clinical context) (**Figure 2B**). In light of these findings, the patient was immediately readmitted to the Department of Oncology for further treatment. Upon admission on April 23, 2022, the patient was administered methylprednisolone sodium succinate [Sinopharm Group Rongsheng Pharmaceutical Co., Ltd., drug registration number: H20030727, specification: 40 mg] at a dose of 80 mg once daily. On April 24, 2022, the dose was adjusted to 80 mg twice daily. Echocardiography results revealed dilatation of the ascending aorta and a decrease in left ventricular diastolic function, with an ejection fraction of 61%. After three days of continuous use, follow-up tests of cardiac injury markers and transaminase showed a decreasing trend. On April 24, 2022, the creatine kinase isoenzyme MB level exceeded 288.00 ng/mL, the myoglobin level was higher than 4126.00 ng/mL, the high-sensitivity troponin I level reached 0.7523 ng/mL, and the creatine kinase isoenzyme MB level was 192.48 ng/mL. The ALT activity was 281.1 U/L, and the AST activity was 675U/L. By April 27, 2022, the cardiac injury marker levels were as follows: creatine kinase isoenzyme at 119.10 ng/mL, myoglobin at 953.00 ng/mL, high-sensitivity troponin I at 0.4797 ng/mL, and creatine kinase isoenzyme MB at 90.36 ng/mL. Meanwhile, the transaminases associated with muscle injury were as follows: ALT at 218.3 U/L and AST at 197 U/L. Given the effectiveness of the hormone therapy, the dose was adjusted to 40 mg twice daily from April 27, 2022, until May 3, 2022. The specific regimen was methylprednisolone sodium succinate 40 mg diluted in 100 mL of 0.9% sodium chloride solution, administered intravenously twice daily. On May 2, 2022, the cardiac injury marker tests indicated the following: creatine kinase isoenzyme at 237.2 ng/mL, myoglobin at 861.5 ng/mL, high-sensitivity troponin I at 0.4193 ng/mL, and creatine kinase isoenzyme MB at 193.78 ng/mL. Additionally, ALT was 147.6 U/L, and AST was 115 U/L. Significant improvements in muscle pain of both lower limbs and back were observed, and the relevant indicators continued to decline. The patient then requested voluntary discharge.

**Figure 3:**
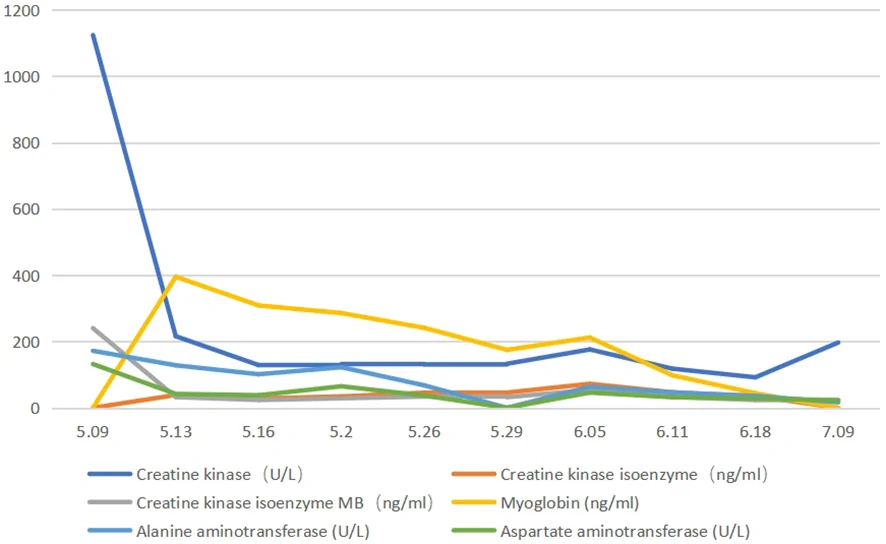
Changes of serological test after methylprednisolone pulse therapy. After methylprednisolone pulse therapy, Creatine kinase, Creatine kinase isoenzyme, Creatine kinase isoenzyme MB, Myoglobin, Alaninaminotransferase and Aspartate aminotransferase indicated decreasing trend.

**Figure 4:**
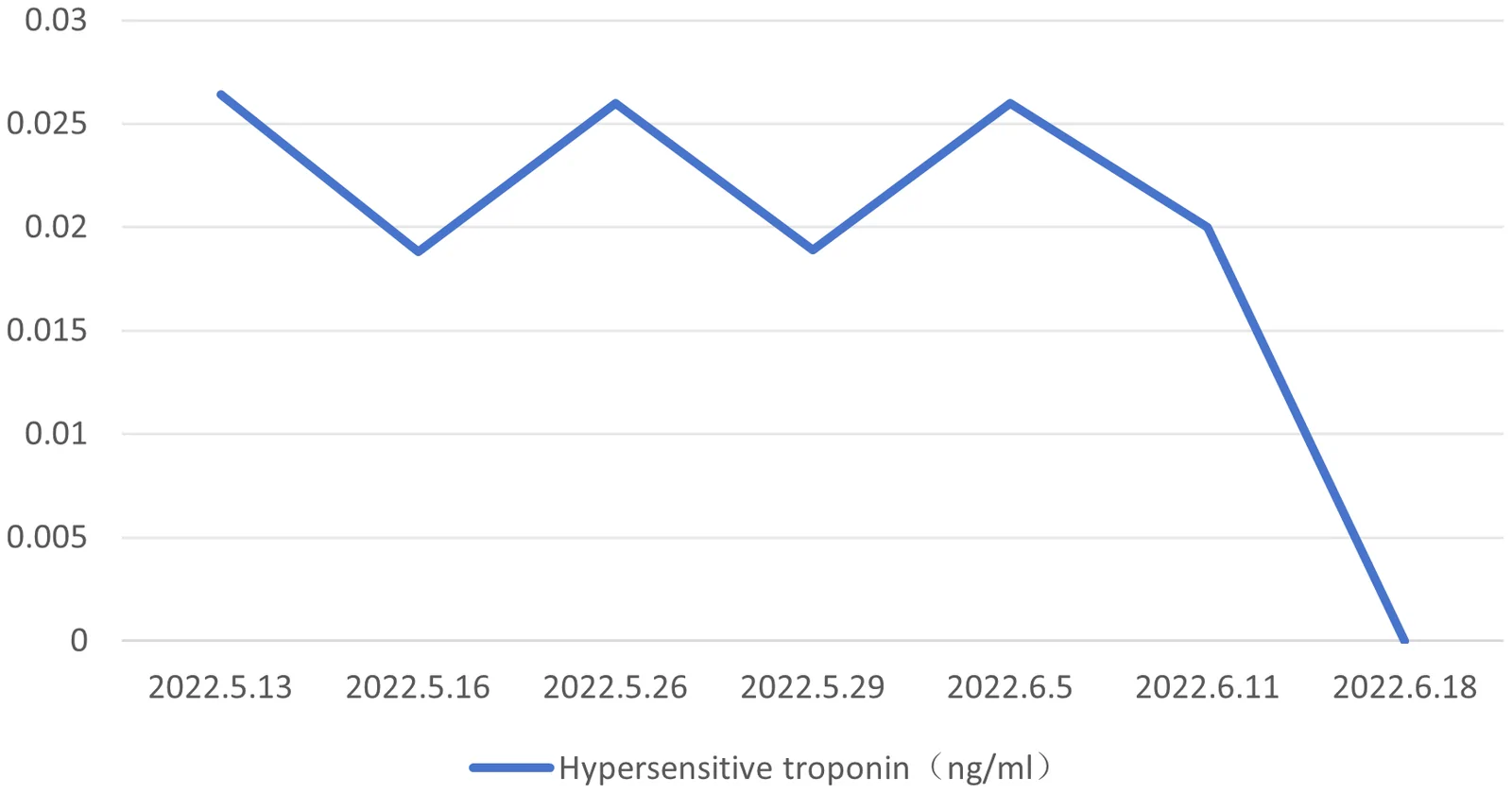
Changes of high-sensitivity cardiac troponin after methylprednisolone pulse therapy. After methylprednisolone pulse therapy, high-sensitivity cardiac troponin indicated a decreasing trend.

After discharge, the patient experienced dizziness upon standing, exacerbated muscle soreness, slurred speech, and later developed coughing while drinking and swallowing weakness. Subsequently, the patient was readmitted to the hospital on May 9, 2022. The ECG showed improvement. Compared with the baseline, the patient’s ECG showed several changes, including arrhythmia, conduction block, STT-T segment changes, T-wave abnormalities, and multi-lead low voltage (**Figure 2C**). Methylprednisolone at a dose of 240 mg once daily and human immunoglobulin at a dose of 20 g once daily were immediately administered. After two days of combination treatment, no significant alleviation of symptoms was observed, and it was suspected that the patient had a steroid-refractory irAE. Therefore, from May 11, 2022, high-dose steroid pulse therapy was initiated, along with human immunoglobulin at a dose of 20 g daily for seven days. The steroid regimen was as follows: Methylprednisolone was initially administered at a daily dose of 1 g for 3 days. Subsequently, the dosage is reduced stepwise (240 mg daily for 3 days, 160 mg daily for 3 days, 80 mg daily for 3 days, 60 mg daily for 3 days, and 40 mg daily for 3 days). After methylprednisolone, the treatment regimen shifts to oral prednisone, initially at 40 mg daily for 7 days, then 20 mg daily for 7 days. During the gradual reduction of steroid dosage, the patient’s lower limb swelling and pain improved, speech gradually became fluent, and no recurrence of coughing while drinking or respiratory difficulties were noted. On May 28, 2022, Chest CT showed that the thymic mass had decreased in size, but the pulmonary nodules remained unchanged (**Figure 1B**). On May 29, 2022, the creatine kinase isoenzyme MB continued to decrease slightly. The patient requested discharge to continue oral prednisone treatment. Outpatient follow-up tests revealed that creatine kinase, creatine kinase isoenzyme, myoglobin, transaminases and high-sensitivity cardiac troponin gradually returned to normal ranges after the discontinuation of oral prednisone (**Figures 3**, **4**). There were no further occurrences of muscle soreness, speech impairment, or coughing while drinking. The patient did not receive further immunotherapy and is currently in a good general condition according to outpatient follow-up. The last follow-up was on May 6, 2024. A formal multidisciplinary team (MDT) discussion was not conducted for this case, as the MDT policy had not been fully implemented in our hospital in 2022. At that time, we adopted a traditional model of multidisciplinary consultation and collective departmental discussion. During treatment, consultations were arranged with relevant departments including Cardiology, Rheumatology and Immunology, Thoracic Surgery, Neurology, and Respiratory and Critical Care Medicine to assist in diagnosis and management.

## Discussion

ICI-related adverse events commonly occur in the skin, colon, endocrine organs, liver, and lungs. Adverse events in the nervous system and cardiovascular system are rare, accounting for only 0.1 - 12%, with complex diagnosis and poor prognosis, which can be life-threatening (12). Among them, neuromuscular diseases are the most common neurological complications in patients receiving ICI treatment, including MG and immune-mediated myopathy, which mostly occur within one month after ICI treatment (6). MG is the most common neuromuscular complication associated with ICI. It is an autoimmune disease mediated by B cells, mainly triggered by autoantibodies against the acetylcholine receptor (AChR) (13). It affects the neuromuscular junction, leading to symptoms of muscle fatigue and weakness, including ptosis, masticatory weakness, dysphagia, dysarthria, difficulty raising the head, limb weakness, etc. In severe cases, the respiratory muscles are involved, resulting in dyspnea and endangering life, with a mortality rate of approximately 30% (14, 15). Makarious et al. reported 23 cases of ICI-related MG, with a mortality rate of 30.4%. Among them, 72.7% were newly diagnosed cases, 18.2% were exacerbations of pre-existing MG, and 9.1% were exacerbations of subclinical MG (16).

Immune checkpoint blockade suppresses negative regulatory pathways (e.g., PD-1/PD-L1), thereby releasing self-reactive T cells. These activated T cells not only attack tumor cells but may also cross-react with shared antigens present in the tumor microenvironment, skeletal muscle, myocardium, and neuromuscular junctions (17, 18). Furthermore, the presence of a thymic mass in this patient likely played a critical role in disrupting central immune tolerance. The thymus is an indispensable organ for T cell maturation; structural or microenvironmental abnormalities within the thymus can lead to aberrant escape of self-reactive T cell clones (19). When combined with the systemic inhibitory effects of sintilimab on immune checkpoints, this created a synergistic environment that substantially amplified the fulminant, multi-organ autoimmune response (20).

MG usually occurs early, and the time from the start of ICI use to the onset of MG symptoms ranges from 6 days to 16 weeks (15). In this study, the patient began to experience symptoms of dysarthria, choking when drinking water, and dysphagia 41 days after ICI treatment, which is consistent with previous reports. MG progresses rapidly after onset. A study reported that the condition of patients with MG caused by nivolumab deteriorated rapidly. Eight severely affected patients showed disease progression within 2–7 days, developed respiratory distress, and rapidly progressed to myasthenic crisis, requiring respiratory muscle support (15). This fully illustrates the importance of timely diagnosis and treatment for MG. The diagnosis of MG requires a comprehensive analysis of typical clinical symptoms, antibody testing [AChR, muscle-specific kinase (MuSK), and Low-density lipoprotein receptor-related protein 4 (LRP4)], and the response to cholinesterase inhibitors (21). Although the case in this study had typical clinical symptoms, there was a lack of corroborating test results. Therefore, we cannot make a definite diagnosis of MG here.

The incidence of ICI-related myocarditis accounts for approximately 0.06%- 3.8% of ICI-related adverse events, while the mortality rate is as high as 39.7% - 50% (22). Therefore, for patients who develop cardiovascular symptoms after ICI treatment and have ECG or cardiac troponin test results, we should be vigilant about the occurrence of myocarditis. ICI-related myocarditis usually occurs within 6 weeks after the first administration of ICI, accompanied by discomfort symptoms such as palpitations, chest tightness, dyspnea, fatigue, and precordial pain. In addition, there will be abnormal ECG findings (ventricular tachycardia, complete heart block, frequent ventricular premature beats, etc.) and elevated cardiac enzymes (mainly troponin I, creatine kinase, and creatine kinase MB). These symptoms may deteriorate rapidly within a few days, potentially leading to cardiogenic shock or cardiac arrest (23, 24). Although the case in this study had typical clinical symptoms, there was a lack of corroborating test results. However, since endomyocardial biopsy is an invasive procedure, it is challenging to implement clinically (25, 26). Therefore, the current clinical diagnosis primarily relies on the European Society of Cardiology (ESC) diagnostic criteria for myocarditis and a history of ICI treatment (27, 28). This patient received ICI treatment, had abnormal ECG, arrhythmia, and elevated troponin. After hormone treatment, the ECG improved, and the troponin level decreased. Therefore, myocarditis is comprehensively considered.

ICI-related myositis is also a rare irAE, but it usually occurs earlier than ICI-related myocarditis and ICI-related MG. Symmetrical proximal limb weakness (such as in the shoulders and pelvic girdle) is the most typical manifestation, often accompanied by muscle pain and fatigue. Some studies have reported that its mortality rate is approximately 21%, and nearly 50% of patients experience prolonged hospital stays or serious complications (29). Currently, the pathogenic characteristics, diagnostic methods, and pathogenesis of ICI-related myositis are not fully understood. It is necessary to routinely measure serum creatine kinase and troponin levels. Muscle biopsy specimens often show extensive infiltration of macrophages in the endomysium and perimysium, as well as the aggregation of CD8^+^, CD4^+^, and CD20^+^ immune cells. Nerve conduction studies, electromyography (EMG), and muscle biopsy are effective means of distinguishing MG from necrotizing myositis (17, 30). ICI-induced myositis and MG exhibit certain overlapping features in clinical presentation, laboratory findings, and electrophysiological characteristics. Differentiating ICI-induced myositis from MG requires a comprehensive assessment integrating clinical manifestations, laboratory tests, electrophysiological studies, and imaging findings. In patients presenting with muscle weakness accompanied by elevated CK levels, negative AChR antibodies, myogenic damage on EMG, and the absence of typical fluctuating muscle weakness, a diagnosis of ICI-induced myositis is more likely (31);Conversely, the presence of fluctuating muscle weakness, positive AChR antibodies, and a decremental response on repetitive nerve stimulation (RNS) supports the diagnosis of MG (32). In clinical practice, these two conditions may coexist, necessitating a multidisciplinary evaluation to formulate individualized treatment plans.

Since immune-related MG, myocarditis, and myositis are triggered by the same abnormal immune response but target different tissues, they may occur simultaneously or successively. Compared to single MG, single myocarditis or myositis, the overlapping immune-related MG, myocarditis, and myositis is even rarer, with more rapid disease progression and a higher mortality rate. It is reported that more than one-third of patients with immune-related MG may also have myositis or myocarditis (33). A study showed that among 12 cases of nivolumab-induced MG, 4 cases were complicated by myositis, 3 by myocarditis, and 1 by both myositis and myocarditis (15). MG patients with concurrent myositis/myocarditis are more likely to develop respiratory failure than those with only MG (54% vs. 42%) (6). Patients with this overlapping syndrome may present with multiple symptoms, and the initial manifestations are often mild, which can easily lead to negligence and misdiagnosis by clinicians. To date, there are no established diagnostic codes or standardized treatment plans for ICI-related MMM overlap syndrome. Therefore, doctors should comprehensively consider the patient’s medical history, clinical manifestations, and the results of various laboratory and diagnostic tests. Given the fulminant course and profound mortality associated with MMM overlap syndrome, it is imperative to establish a rigorous, multidisciplinary diagnostic framework in clinical practice. When patients subjected to ICI therapy present with overlapping symptoms such as ptosis, dysphagia, dyspnea, and proximal muscle weakness, a stepwise diagnostic approach must be promptly initiated. Initial evaluation should simultaneously assess cardiac stress biomarkers (high-sensitivity troponin and natriuretic peptides), muscle enzymes (creatine kinase and aldolase), and a specific autoantibody panel (including AChR and MuSK antibodies). Subsequently, electrophysiological assessments, such as EMG and RNS, should be rapidly performed to distinguish myogenic injury from neuromuscular junction damage. Furthermore, cardiac MRI is strongly recommended to detect myocardial edema and late gadolinium enhancement. Most importantly, we emphasize that, given the rapid and often fatal progression of the MMM triad (18), empiric high-dose corticosteroid therapy—combined with intravenous immunoglobulin (IVIG) or plasmapheresis when indicated—must be initiated immediately once clinical suspicion is high, without delay while awaiting confirmatory diagnostic results. If patients receiving ICI present with symptoms such as exertional dyspnea, dysphagia, diplopia, dysarthria, limb weakness, or inability to walk due to muscle pain, this overlapping syndrome should be highly suspected. For patients with severe conditions, such as complete heart block (CHB) or malignant arrhythmias, vital signs should be monitored in real time, and they should be admitted to the intensive care unit if necessary and managed by a multidisciplinary team (34–36).

Current guidelines for ICI-related adverse events recommend discontinuing ICI and initiating corticosteroid therapy, and high-dose corticosteroid pulse therapy if necessary. If the patient shows signs of improvement within 24 hours, the corticosteroid dose can be gradually reduced over at least 4 weeks (37, 38). For refractory cases, combination therapy with other immunosuppressants may be required, including IVIG, infliximab, and antithymocyte globulin. It is worth noting that infliximab may exacerbate heart failure, so it should not be used in patients with moderate to severe heart failure (39). The patient in this study is the first case in our hospital to present with overlapping immune myocarditis, immune myositis, and symptoms of MG after receiving ICIs treatment. The patient was initially diagnosed with myocarditis stage G2. According to the Chinese Society of Clinical Oncology guidelines for the management of immune checkpoint blocker-related toxicities (2021) and prior treatment experience, methylprednisolone was administered at a dose of 1–4 mg/kg/day for five consecutive days, followed by gradual tapering. Due to an inadequate response to this regimen, high-dose corticosteroid therapy combined with immunoglobulin was initiated, after which the patient’s symptoms gradually resolved. We propose that the disease rebound may have resulted from a combination of premature dose tapering and the inherently aggressive nature of the MMM triad. Immune checkpoint blocker (ICB)-related MMM overlap syndrome is associated with extremely high mortality and progresses more rapidly than primary autoimmune diseases (36, 40). For patients presenting with or progressing to the MMM triad, we recommend initiating high-dose “pulse” corticosteroid therapy combined with IVIG from the outset (18, 35), rather than escalating treatment only after standard doses have proven ineffective. Fortunately, the patient did not experience respiratory muscle.

In addition, combined with CT examination, there was a thymic mass in this case, and it significantly shrank after anti - tumor treatment. However, because no pathological diagnosis was obtained, the nature of the lesion cannot be determined. Thymoma and thymic metastasis of colon cancer may exhibit overlapping imaging and pathological features. When both lesions are present, pathological biopsy, immunohistochemistry, and genetic testing (e.g., microsatellite instability [MSI] testing) are essential to establish a definitive diagnosis, thereby preventing misdiagnosis as a single disease entity and ensuring targeted treatment plans. In patients with primary thymoma, underlying “autoimmune deficiency” commonly develops prior to immunotherapy. Treatment with ICIs can lead to rapid expansion of autoreactive immune cells and early-onset, severe irAEs, including myositis, myocarditis, hepatitis, and MG, occurring within 1 to 6 weeks (41, 42), with an incidence rate of 58.3% (40). Therefore, thymoma confers a high baseline risk of immunotoxicity associated with ICI use. Colon cancer with MSI-H that metastasizes to the thymus may locally induce a robust inflammatory microenvironment remodeling (43), This disruption of the local microenvironment, combined with systemic T-cell activation induced by ICIs, may generate a synergistic effect, ultimately triggering a cross-reactive immune attack targeting the myocardium, skeletal muscles, and neuromuscular junctions. Therefore, we suspect that the thymic mass may have contributed to the development of the overlapping syndrome in this patient. Of course, this requires further research for confirmation. This reminds us that when performing ICI therapy in patients with thymic masses, we need to be particularly vigilant for immune-related complications.

In conclusion, MG, myositis, and myocarditis caused by ICIs are rare but fatal. Doctors need to comprehensively consider the patient’s symptoms, signs, and examination results and be vigilant about the occurrence of these complications, especially the overlapping triad. In particular, for patients with thymic masses, we need to be more vigilant for immune-related adverse reactions and promptly implement active treatment measures.

## Data Availability

The original contributions presented in the study are included in the article/**Supplementary Material**, further inquiries can be directed to the corresponding author/s.

## Supplementary material

Supplementary Material 1 Laboratory parameters and tumor marker results of the patient.
